# Extracellular Vesicle and Plasma miRNAs as Candidate Biomarkers of Traumatic Brain Injury in the Context of Polytrauma

**DOI:** 10.3390/ijms27104248

**Published:** 2026-05-10

**Authors:** Cora Rebecca Schindler, Dirk Henrich, Lena Krämer, Inna Schaible, Jason-Alexander Hörauf, Aileen Ritter, Philipp Störmann, Rald Victor Maria Groven, Markus Huber-Lang, Ingo Marzi, Liudmila Leppik

**Affiliations:** 1 Department of Trauma Surgery and Orthopedics, University Hospital of Goethe University Frankfurt, 60323 Frankfurt am Main, Germany; 2 Department of Trauma and Reconstructive Surgery, University Hospital RWTH Aachen, 52074 Aachen, Germany; 3 Institute of Clinical and Experimental Trauma-Immunology, University Hospital of Ulm, 89081 Ulm, Germany

**Keywords:** traumatic brain injury, extracellular vesicles, biomarkers, miRNA, polytrauma

## Abstract

Severe traumatic brain injury (TBI) is a leading cause of mortality and long-term disability in polytrauma (PT) patients, and its clinical outcome remains difficult to predict due to clinical heterogeneity and secondary injury mechanisms. Current diagnostic and prognostic approaches based on clinical assessment and imaging are limited, particularly in PT where neurological evaluation is often impaired. This study aimed to compare plasma- and extracellular vesicle (EV)-associated microRNA (miRNA) signatures in patients with severe TBI and healthy controls to identify their potential as minimally invasive biomarkers and to improve understanding of molecular responses. For profiling circulating miRNAs, blood samples were collected at ≤3 h and at 48 h after admission. In the screening phase, plasma samples of *n* = 15 patients with severe isolated TBI (Abbreviated Injury Scale [AIS]Head ≥ 4, all other AIS ≤ 1) and *n* = 15 age- and sex-matched healthy controls were pooled (*n* = 5/pool) and subjected to next-generation sequencing (NGS). In the following validation phase, *n* = 25 severely injured trauma patients (Injury Severity Score [ISS] ≥ 16) were enrolled and stratified into PT without TBI (PT; AISHead = 0; *n* = 13) and isolated TBI (*n* = 12). Differentially expressed candidate miRNAs identified in the screening phase were validated in individual plasma and EV samples using reverse transcription droplet digital polymerase chain reaction (RT-ddPCR). Functional enrichment and pathway analyses were performed using miRNet. NGS identified more differentially expressed miRNAs in plasma (ER: 103; 48 h: 65) than in EVs (Emergency Room [ER]: 14; 48 h: 32). Functional enrichment analysis indicated associations with pathways related to cellular stress, senescence, growth factor signaling, transcriptional regulation, and apoptosis. In validation, 12 of 16 plasma and 10 of 15 EV-miRNAs were confirmed as differentially expressed in TBI patients; among these, three plasma and four EV miRNAs differed between TBI and PT. After adjustment, most plasma miRNAs were associated with injury severity rather than group status. EV miRNA profiles showed heterogeneous patterns, with miR-1469 associated with TBI group status in adjusted analysis, while miR-1237-5p was linked to injury severity and other EV miRNAs showed no consistent group-specific effects. Plasma miRNAs mainly correlated with systemic injury markers, whereas EV miR-1469 showed a moderate association with the Glasgow Coma Scale (GCS). Overall, circulating miRNA profiles after injury appear to be predominantly influenced by systemic trauma severity rather than TBI-specific effects. Plasma miRNAs mainly reflected general injury burden, whereas EV-associated miRNAs showed more heterogeneous patterns, with miR-1469 emerging as a candidate associated with TBI after adjustment for clinical covariates. These findings suggest that EV-derived miRNAs, particularly miR-1469, may provide more targeted signals related to brain injury and warrant further investigation.

## 1. Introduction

Severe traumatic brain injury (TBI) is a major cause of mortality and long-term disability following polytrauma (PT) in adults worldwide [1,2,3,4]. Despite advances in critical care medicine, clinical outcome after TBI remains highly variable and difficult to predict. The complex pathophysiology of TBI involves an initial mechanical insult followed by secondary injury mechanisms [5], including neuroinflammation, oxidative stress, excitotoxicity, and disruption of the blood–brain barrier (BBB), which evolve over time and contribute to progressive neuronal damage [6,7]. Current diagnostic and prognostic tools, primarily based on clinical assessment and radiological imaging [8], are of limited value in reflecting ongoing pathophysiological processes, especially in PT patients [9,10,11], where multiple injuries, sedation, and mechanical ventilation impede reliable neurological assessment [12,13,14].

Blood-based biomarkers can serve as minimally invasive tools to improve diagnostic accuracy and outcome prediction. Protein biomarkers for TBI, such as S100 calcium-binding protein B (S100B) and glial fibrillary acidic protein (GFAP) have been proposed [15,16,17]; however, their clinical utility is limited by low specificity due to extracranial expression, particularly in patients with PT [18].

MicroRNAs (miRNAs) represent a promising alternative class of biomarkers. These small, non-coding RNA molecules regulate gene expression at the post-transcriptional level and are involved in key biological processes [19,20,21], including (neuro)inflammation [22], apoptosis, and neuronal plasticity [23]. Following TBI, miRNA expression profiles are altered in response to tissue damage and secondary injury cascades [24]. MiRNAs are stable in peripheral blood and can cross the (disrupted) BBB [25], either as freely circulating molecules or encapsulated within extracellular vesicles (EVs) [26,27]. EVs are actively released by cells, carry bioactive cargo such as proteins and miRNAs and play a central role in intercellular communication [28,29]. EV-associated miRNAs are protected from enzymatic degradation and may more specifically reflect neuro-pathophysiological processes than freely circulating miRNAs [30]. Recently, we and others have reported that injury-associated miRNAs are detectable in plasma, EVs, and neuron-derived EVs, and that their expression profiles differ depending on both the biological compartment and the underlying injury pattern [31,32,33,34,35]. Moreover, emerging evidence suggests that specific EV subpopulations, characterized by surface markers such as Myelin Oligodendrocyte Glycoprotein (MOG^+^) or Aminopeptidase N (CD13^+^), may be enriched in TBI and correlate with clinical neurological status [36].

Based on our previous studies, we hypothesize that freely circulating and EV-associated miRNAs in the plasma of adult TBI patients show distinct expression profiles and provide complementary diagnostic and prognostic information. Using next-generation sequencing (NGS), this study aims to systematically compare miRNA signatures in plasma and plasma-derived EVs to identify novel biomarkers and gain insight into the molecular response to severe TBI.

## 2. Results

**Figure 1:**
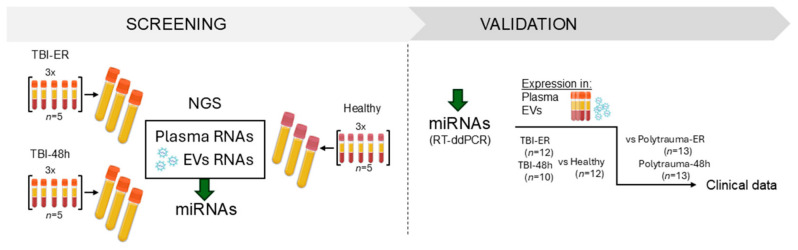
Study design. The study was divided into two phases: a screening phase and a validation. In the screening phase, 15 severely injured trauma patients (injury severity score [ISS] ≥ 16) with severe isolated traumatic brain injury (TBI, Abbreviated Injury Scale [AIS] Head ≥ 4, all other AIS ≤ 1) and 15 age- and sex-matched healthy volunteers (Healthy) were included. Plasma from five patients (or controls) were pooled and used for miRNA and extracellular vesicle (EV) isolation, followed by EV-miRNA extraction. In total, nine pooled plasma samples for miRNA and EV-associated miRNA analysis were processed for next-generation sequencing (NGS). In the validation phase, 25 severely injured trauma patients (ISS ≥ 16) were allocated to polytrauma (PT, AISHead = 0; *n* = 12) and TBI (*n* = 13) cohorts and included together with 12 healthy controls. Differential expression of selected miRNAs was validated in plasma and EVs using reverse transcription droplet digital polymerase chain reaction (RT-ddPCR), first in the TBI cohort and then in the PT cohort.

For the initial screening via NGS 15 severely injured trauma patients (injury severity score [ISS] ≥ 16) with severe isolated TBI (TBI, Abbreviated Injury Scale [AIS]Head ≥ 4) and 15 age- and sex-matched healthy volunteers (controls) were included (Figure 1). For validation we included 12 TBI patients, 13 PT patients, and 12 healthy volunteers as controls.

**Figure 2:**
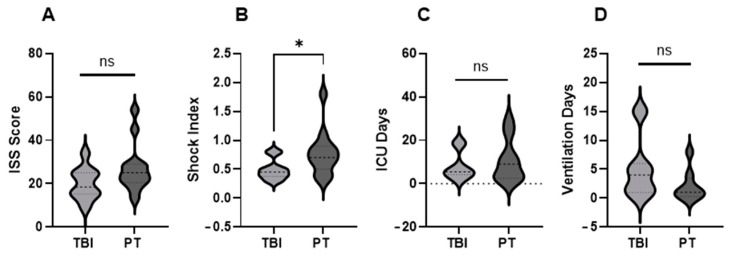
Demographic and clinical characteristics of the PT and TBI cohorts. (**A**) The median injury severity score (ISS) was higher in PT patients (25 [IQR 21–29]) than in TBI patients (19 [IQR 16–22]), although the difference was not statistically significant. (**B**) Shock index (SI) differed between the groups. SI categories were defined as <0.6 (no shock), 0.6–1.0 (mild), 1.0–1.4 (moderate), and ≥1.4 (severe). In the TBI cohort, 10 patients had no shock and 2 had mild shock. In the PT cohort, 5 patients had no shock, 6 had mild shock, 1 had moderate shock and 1 had severe shock. (**C**) PT patients had a longer length of stay in the intensive care unit (ICU). (**D**) TBI patients accumulated more ventilations days. Violin plots show the distribution of variables across groups. The width represents data density. The dashed line indicates the median, and the inner lines represent the quartiles. * *p* < 0.05. ns = not significant.

The demographic and clinical data for the TBI and PT cohorts are presented in Figure 2 and Supplementary Table S1. The average ISS score of the TBI cohort (isolated injury) was lower than that of the PT cohort; however, it was not significant (Figure 2). The shock index (SI), defined as the ratio of heart rate (HR) to systolic blood pressure (SBP) (SI = HR/SBP), was used to assess hemodynamic instability. In the TBI cohort two patients showed mild signs of shock. In the PT cohort, six patients exhibited mild SI, and one patient showed moderate to severe shock. Although patients in the TBI group required longer periods of mechanical ventilation, PT patients required prolonged intensive care unit (ICU) treatment.

**Figure 3:**
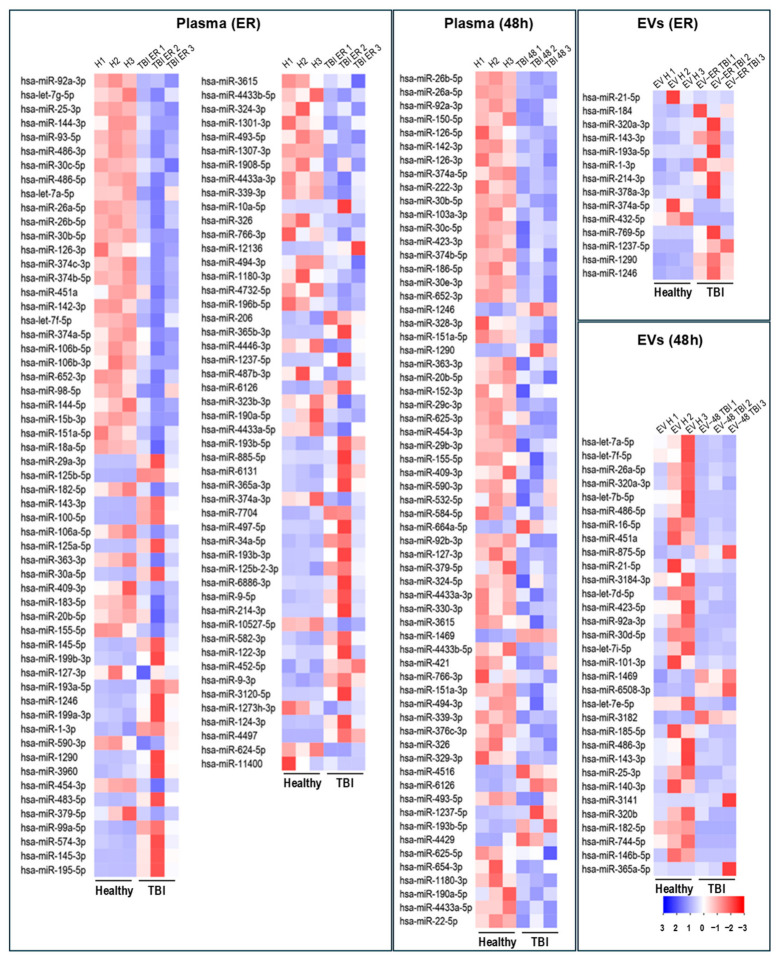
Overview of differentially expressed miRNAs in TBI patients versus healthy controls at ER and 48 h in plasma and EVs, shown as heat maps. Heat maps illustrate expression profiles of miRNAs in pooled plasma and EV samples from patients with isolated TBI at emergency room (ER) admission and at 48 h compared with healthy controls. Data are scaled for each row (each miRNA); red indicates upregulated and blue indicates downregulated expression (fold change). H1, H2, and H3—pooled samples from healthy control participants; TBI ER (48 h) 1, 2, and 3–pooled samples from TBI patients, collected either at ER admission or at 48 h post-injury.

NGS was performed to compare plasma- and EV-associated miRNA profiles in the emergency room (ER, ≤3 h after injury) and after 48 h (capturing primary and secondary brain injury) in TBI patients and healthy controls. The number of miRNAs identified as differentially expressed in patients was higher in plasma (ER: 103 miRNAs; 48 h: 65 miRNAs) than in EVs (ER: 14 miRNAs; 48 h: 32 miRNAs) (Figure 3). Most miRNAs were downregulated after TBI in both plasma and EVs, except for EV-miRNAs at ER admission.

To further analyze the functional relevance of the differentially expressed miRNAs, explorative miRNA network and Reactome-based pathway and biological process enrichment analyses were performed using miRNet (Table 1, Supplementary Table S2). Table 1 shows the Top 10 enriched pathways based on in silico-predicted target genes. Pathway enrichment analysis revealed a strong association with cellular stress and senescence programs (oxidative stress- and oncogene-induced senescence), growth factor-mediated signaling (PDGF, NGF, TGF-β, SCF-KIT), and regulation of gene expression and apoptosis, including NOTCH-related transcriptional processes.

Biological process enrichment analysis (Supplementary Table S2) highlighted processes involved in transcriptional and translational repression, downregulation of metabolic and biosynthetic pathways, regulation of cell cycle progression and protein kinase activity, and DNA damage response signaling, particularly through p53-class mediators.

**Figure 4:**
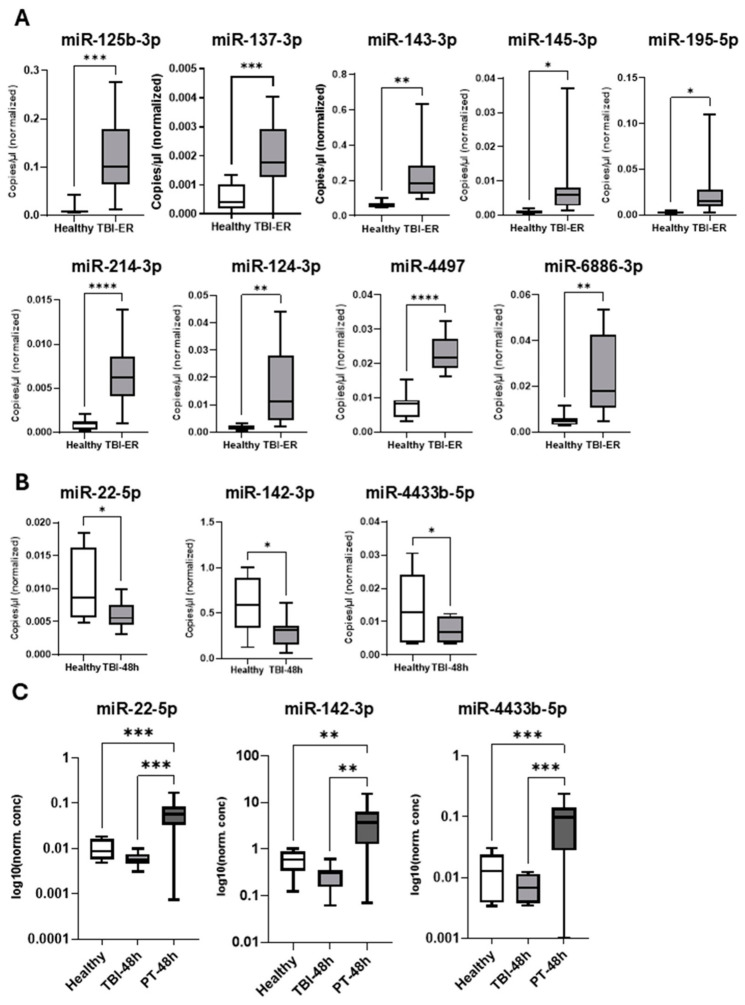
Validation of plasma-miRNA expression data obtained by NGS using RT-ddPCR. (**A**): plasma-miRNA miR-125b-3p, miR-137-3p, miR-143-3p, miR-145-3p, miR-195-5p, miR-214-3p, miR-124-3p, miR-4497 and miR-6886-3p were significantly upregulated at the time of admission to the emergency room (ER) compared with healthy controls. (**B**): miR-22-5p, miR-142-3p and miR-4433b-5p showed significantly lower plasma levels compared with healthy volunteers at 48 h following TBI. (**C**): TBI association analysis (healthy vs. TBI vs. PT). At 48 h, plasma levels of miR-22-5p, miR-142-3p and miR-4433b-5p were significantly lower in TBI compared with PT and showed a trend toward lower levels compared with healthy controls. * *p* < 0.05; ** *p* < 0.01; *** *p* < 0.001; **** *p* < 0.0001.

**Figure 5:**
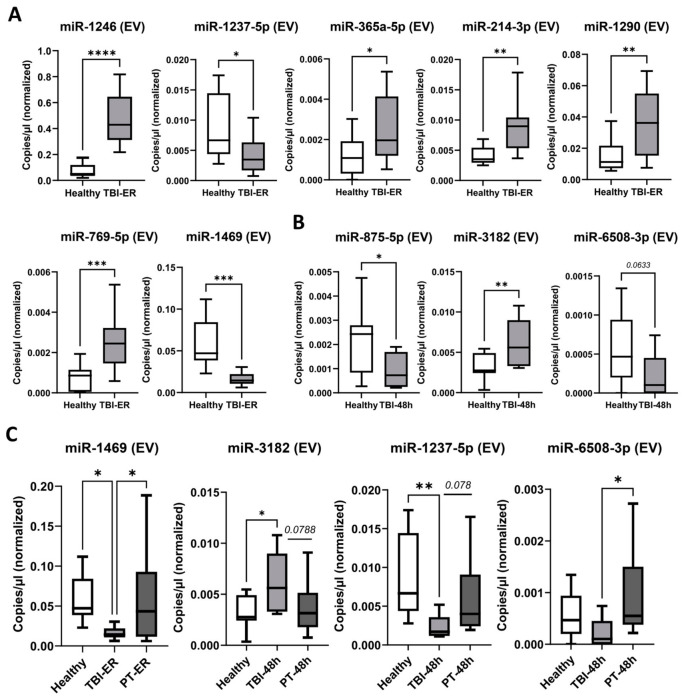
Plasma-EV-miRNA expression analysis via RT-ddPCR. (**A**): At the time of admission to the emergency room (ER) EV-miRNAs miR-1246, miR-365a-5p, miR-214-3p, miR-1290 and miR-769-5p were upregulated whereas miR-1237-5p, miR-1469 were downregulated in TBI patients compared to healthy volunteers. (**B**): At 48 h post-TBI, EV-associated miR-875-5p and miR-6508-3p levels were significantly lower, whereas miR-3182 levels were significantly higher in TBI patients compared with healthy controls. (**C**): TBI association analysis (healthy vs. TBI vs. PT): EV miR-1469 was significantly downregulated within ≤3h after TBI. At 48 h post-TBI, miR3182 was upregulated, while miR-1237-5p showed a trend towards downregulation compared with the PT cohort. miR-6508-3p was significantly downregulated compared with PT and showed a trend toward lower levels in healthy controls. * *p* < 0.05; ** *p* < 0.01; *** *p* < 0.001; **** *p* < 0.0001.

Out of all differentially expressed miRNAs in TBI patients, we selected miR-125b-3p, miR-137-3p, miR-143-3p, miR-145-3p, miR-195-5p, miR-214-3p, miR-124-3p, miR-4497, miR-6886-3p, miR-22-5p, miR-142-3p, miR-4433b-5p, miR-1469, miR-376 and miR-339 from plasma for validation analysis, based on the highest differential expression and/or evidence given in the literature. From EV-miRNAs, we selected miR-1246, miR-3182, miR-374a-5p, miR-423-5p, miR-365a-5p, miR-1469, miR-214-3p, miR-1290, miR-875-5p, miR-184, miR-6508-3p, miR-1237-5p, miR-769-5p, miR-486-5p and miR-92a-3p (Figure 4 and Figure 5).

Expression of these miRNAs was assessed via RT-ddPCR in TBI patients and healthy controls and compared. Out of the 16 tested plasma miRNAs 12 were confirmed to be differentially expressed in TBI patients (Figure 4A,B) and proceeded for further analysis. At this step their expression was compared between TBI, PT and healthy controls. Figure 4C shows significant decrease in expression of miR-22-5p, miR-142-3p and miR-4433b-5p in TBI patients compared with PT and a trend towards decreased expression compared with controls.

Among 15 selected dysregulated EV miRNAs, 10 were validated (Figure 5A,B) and further analyzed in PT patients, with comparisons to TBI patients and healthy controls (Figure 5C). EV-associated miRNA profiles showed more distinct patterns in TBI, with significantly decreased levels of miR-1469 (TBI < control and PT, *p* < 0.05) and miR-1237-5p (TBI < control *p* < 0.01, trend vs. PT *p* = 0.078). MiR-3182 was increased in TBI (TBI > control *p* < 0.05, >PT *p* = 0.0788), whereas miR-6508-3p was decreased compared with PT (TBI < PT, *p* < 0.05).

Considering the differences between the TBI and PT groups in terms of age, injury severity score (ISS) and ASA classification (American Society of Anesthesiologists Physical Status Classification System) (Supplementary Table S1), plasma- and EV-derived miRNA expression was analyzed in TBI patients, PT patients, and healthy controls using multiple linear regression adjusted for group, age, ISS, and ASA classification. In plasma, miR-22-5p, miR-142-3p, and miR-4433b-5p showed no independent association with group after adjustment for covariates. However, significant associations with ISS were observed for miR-22-5p (β = 0.0069, *p* = 0.0011) and miR-4433b-5p (β = 0.0044, *p* = 0.0041), indicating a relationship with injury severity. miR-4433b-5p was additionally associated with age (β = −0.0018, *p* = 0.0364) (Supplementary Table S3).

Among EV-derived miRNAs, miR-1469 was significantly associated with group status (β = −0.0716, *p* = 0.0111) after adjustment for age, ISS, and ASA, whereas no association with ISS or other covariates was observed. EV miR-1237 showed a significant association with ISS (β = 0.00061, *p* = 0.0034), while EV miR-3182 showed no significant associations. EV miR-6508 did not show any significant associations with group, age, ISS, or ASA.

Overall, most miRNAs were associated with injury severity rather than group status, with the exception of EV miR-1469, which showed an independent association with the TBI group.

To investigate the potential clinical and biological significance of miRNA dysregulation, expression levels of plasma- and EV-associated miRNAs differentially expressed between TBI and PT were correlated with clinical and organ-specific parameters of these patients (Table 2). In the plasma, miR-142-3p, miR-22-5p, and miR-4433b-5p were significantly upregulated in PT compared with TBI and healthy controls. Table 2 shows significant moderate correlations of these miRNAs with systemic clinical and laboratory parameters of (organ) perfusion, bleeding and coagulation, including thrombocyte count (miR-142-3p: r = 0.49, *p* = 0.02; miR-4433b-5p: r = 0.45, *p* = 0.03), base excess ([BE], miR-142-3p: r = −0.45, *p* = 0.04), or SI (miR-4433b-5p: r = 0.43, *p* = 0.05). miR-4433b-5p shows organ-specific correlations with liver-related biomarkers, including aspartate aminotransferase (AST) and glutamate dehydrogenase (GLDH), (r = 0.43, *p* = 0.04; and r = 0.5, *p* = 0.01). Additionally, we found a significant link with creatine kinase ([CK], miR-4433b-5p and miR-22-5p) and myoglobin (miR-4433b-5p) that implicates a musculoskeletal trauma that occurs quite ubiquitously in PT.

In contrast, EV-associated miRNAs (miR-1469-3p, miR-1237-5p and miR-6508-3p), which differed between TBI, PT and healthy controls, showed variable associations with injury-related clinical parameters. Downregulated EV-associated miR-1469 showed a moderate positive correlation with Glasgow Coma Scale ([GCS], r = 0.43, *p* = 0.05) and a strong positive correlation with 48 h C-reactive protein (CRP) levels (r = 0.76, *p* = 0.04). The downregulated EV-miR1237-5p was associated with pro-inflammatory Interleukin (IL)-6 48 h post-injury (r = 0.55, *p* = 0.01). Downregulated miR-6508-3p showed significant correlations with overall injury severity as assessed by the ISS (r = 0.44, *p* = 0.04). Both miR-3182 and miR-6508-3p also correlate with clinical parameters of multiple organ dysfunction (Aspartate-Aminotransferase (ALT), Myoglobin, and Creatine kinase (CK)).

## 3. Discussion

In the present study we hypothesized that freely circulating and EV-associated miRNAs in plasma of adult TBI patients exhibit distinct expression profiles and provide complementary diagnostic and prognostic information and therefore could serve as biomarkers for detecting TBI in the complex context of PT. To test this hypothesis, dysregulated miRNAs in TBI patients were characterized in two distinct plasma compartments—freely circulating miRNAs and EVs—and compared across different trauma cohorts. Two time points (admission to the emergency room and 48 h post-injury) were selected to capture both early injury responses and later secondary neuroinflammatory processes, enabling detection of dynamic miRNA changes.

### 3.1. miRNA Profiles

Our miRNA profiling data indicate that, although both compartments exhibit predominantly downregulated miRNA sets at both time points, the extent and number of dysregulated miRNAs differ, with a greater number observed in plasma compared to EVs. Pathway enrichment analysis also revealed distinct differences between plasma and EV miRNA profiles. At the early response (ER) time point, miRNA upregulated in plasma—therefore predicted to suppress their target genes—were primarily associated with stress response, growth factor-mediated survival, and regulated cell death pathways. Enrichment of NOTCH and senescence-related pathways suggests early adaptive and differentiation-related regulatory programs. In contrast, EVs showed upregulation of miRNA targeting pathways involved in metabolic adaptation (including glucose transporter (GLUT)-4 signaling), angiogenesis, and proliferative signaling, indicating selective vesicle-associated regulation of metabolic and growth-related processes. MiRNAs downregulated in both plasma and EVs—implying de-repression of their targets—were associated with NOTCH signaling, transforming growth factor (TGF)-β signaling, and senescence pathways, suggesting a systemic shift toward increased proliferation, differentiation, and stress-response activity. Notable compartment-specific differences were also observed. In plasma, downregulated miRNAs were mainly linked to broad transcriptional regulation and senescence control, whereas in EVs, downregulated miRNAs additionally targeted extrinsic apoptosis and toll-like receptor 4 (TLR4)-mediated immune response pathways, pointing to EV-specific modulation of immune signaling and cell death programs. At the 48 h time point, both plasma and EV compartments exhibited widespread miRNA changes predicted to suppress transcriptional activity, stress-response programs, senescence, and NOTCH/ Stem Cell Factor/c (SCF)–KIT signaling, indicating a global dampening of adaptive and proliferative responses at this later stage. Compartment-specific differences were also evident. In plasma, regulatory effects were largely confined to general transcriptional control and senescence pathways, whereas EVs showed additional suppression of immune-related signaling, including B-cell receptor pathways, highlighting temporally regulated, compartment-specific modulation of immune activity. Overall, these findings suggest a dynamic temporal shift in miRNA-mediated regulation following injury in both compartments.

### 3.2. miRNA Expression Patterns Across Polytrauma and TBI Groups

Results of the validation study indicate that the selected miRNAs upregulated in TBI plasma are predominantly associated with a general injury response, as comparable or higher expression levels were observed in PT patients. A similar pattern was seen for EV-associated miRNAs upregulated in TBI, except for miR-3182, which showed a trend toward increased expression in TBI compared with PT. These findings suggest that miRNAs showing injury-induced upregulation are likely not restricted to TBI within the context of PT.

In contrast, miRNAs downregulated in TBI appeared to show a more distinct pattern. In plasma, all tested TBI-downregulated miRNAs were upregulated in PT patients, whereas EV-associated miRNAs showed no significant changes between PT and controls. Notably, most downregulated miRNAs were observed at 48 h, a phase relevant to secondary injury and physiological adaptation, suggesting divergent temporal expression trajectories between TBI and PT.

Overall, plasma miRNAs were more strongly associated with systemic injury severity rather than TBI-specific effects. In particular, plasma miR-22-5p and miR-4433b-5p were significantly associated with ISS, supporting a predominant influence of overall trauma burden. According to the literature, miR-22-5p has traditionally been considered the passenger strand of miR-22 and thought to undergo degradation. While our findings do not support a strong TBI association, miR-22-5p was downregulated 48 h after TBI and showed correlations with clinical parameters such as RISC2, creatine kinase and vasopressor use. Together with emerging evidence implicating miR-22-5p in cardiovascular and oncological pathways [37,38], these observations suggest a potential functional role in pathophysiological processes and warrant further investigation.

miR-142-3p is highly abundant in cells of hematopoietic origin, and its role in the regulation of immune response has received major attention [39]. Early studies proposed this miRNA as a potential molecular marker for assessing TBI progression based on temporal changes in its expression following TBI [40,41]. In the present study miRNA-142-3p was downregulated 48 h after TBI; however, it did not show an independent association with group status after adjustment for age, ISS, and ASA. These findings suggest that observed changes in miR-142-3p expression may be influenced by injury-related clinical factors rather than representing a TBI-specific effect.

We identified correlations of miR-4433b-5p with clinical parameters related to shock, hemorrhage, and coagulation disturbances. In our study, miR-4433b-5p was significantly associated with ISS, supporting a predominant influence of overall trauma burden. To date, only limited data are available on this miRNA, with previous studies mainly linking it to inflammatory conditions, including autoimmune diseases [42] and COVID-19 [43], as well as its potential role as a tumor-associated biomarker [44]. Exosomal miR-4433b has also been previously associated with neurodegenerative disorders, including Alzheimer’s and Parkinson’s disease [30], where involvement in mitochondrial dysfunction and oxidative phosphorylation has been suggested [45]. Its role in trauma remains largely unexplored; however, it is plausible that miR-4433b-5p may contribute to post-traumatic inflammatory responses.

In contrast, EV-associated miRNAs showed a more heterogeneous pattern. EV miR-1469 was independently associated with TBI group status after adjustment for ISS, age, and ASA classification, and showed a moderate correlation with GCS. miR-1469 has primarily been described in the context of cancer-related studies [46,47], where it has been investigated mainly as a potential circulating biomarker. However, its functional role in EV-mediated intercellular communication remains largely uncharacterized. Recently miR-1469-containing exosomes were shown to enhance NK cell proliferation and increase IFN-γ secretion following uptake [48], suggesting immunomodulatory effects. In our study, EV miR-1469 showed differential expression between TBI, PT, and healthy controls and remained associated with TBI group status in adjusted analysis, suggesting a potential TBI-associated EV signal that warrants further validation.

Three other EV-associated miRNAs showed heterogeneous associations with clinical parameters. EV miR-1237 was significantly associated with ISS and showed correlations with the Glutamate Dehydrogenase (GLDH) and IL-6, suggesting a potential link to systemic injury severity. It has been previously described mainly in cancer-related profiling studies, with limited functional evidence and emerging but still sparse reports in neurological contexts [49,50]. EV miR-3182, a poorly characterized miRNA identified primarily through sequencing-based studies, showed correlations with markers of tissue injury such as creatine kinase and myoglobin but no consistent associations with group status or other clinical covariates. EV miR-6508, also largely uncharacterized, showed correlations with several biochemical parameters including ISS-related and organ injury markers (e.g., CK, GLDH, ALT, hemoglobin), but no significant association with group or other covariates.

### 3.3. Clinical Relevance

Overall, plasma miRNAs were more strongly associated with general injury severity, whereas EV-associated miRNAs showed more heterogeneous patterns, with EV miR-1469 retaining a group-related signal in adjusted analysis. These findings suggest differential regulatory patterns between plasma- and EV-derived miRNAs in trauma. This may be attributable to the predominantly passive release of miRNAs into plasma from damaged tissues and their susceptibility to systemic influences in PT, which can obscure organ-specific signals.

In contrast, EV-associated miRNAs are actively and selectively loaded into EVs through regulated mechanisms and are thereby protected from degradation, which may allow them to more reliably reflect cell-type-specific activation states and ongoing biological processes. This is consistent with previous trauma studies, reporting compartment-specific miRNA responses, in which EV-associated—particularly neuron-derived EVs—miRNAs display delayed yet more TBI-specific signatures, while total plasma miRNA profiles are influenced earlier and more strongly by systemic factors [51,52,53]. In addition, EV-associated miRNAs may reflect not only primary injury but also secondary brain injury processes such as neuroinflammation, tissue repair, and glial activation, which are difficult to assess clinically in sedated or mechanically ventilated PT patients due to limited neurological evaluation.

Future biomarker studies in TBI may benefit from focusing on EV-derived miRNAs, which appear less affected by systemic injury-related confounding than plasma miRNAs in this cohort. In particular, downregulated EV-associated miRNAs may represent promising candidates for TBI-related signal detection.

### 3.4. Study Limitations

Due to the small number of patients with isolated TBI who met the inclusion criteria of the study, only *n* = 15 (12) per group were included. The relatively low number of patients represents a limitation, as individual variability in clinical parameters and diagnostic measures may disproportionately influence the results. In addition, inherent differences in age (older TBI patients) and injury severity (lower ISS in isolated TBI compared with PT), which reflect real-world clinical conditions, likely influenced the observed findings and contributed to the attenuation of group differences after multivariable adjustment. This reduces statistical power, limits the detection of subtle differences, and restricts the generalizability of the findings.

From a methodological perspective, the pooling strategy used during the identification phase was intended to reduce the impact of individual variability and highlight common patterns. However, this approach may have limited the sensitivity of detection and could have led to the omission of low-abundance miRNAs, potentially overlooking some biologically relevant signals. The relatively small volume of patient plasma available for analysis represents a further limitation, as it may have constrained the sensitivity of NGS and PCR, potentially leading to underrepresentation of low-abundance miRNAs.

Although established biomarkers such as GFAP and S100B were not included for direct comparison due to their known limitations in PT [18], future studies integrating these markers with miRNA-based approaches may further improve diagnostic accuracy. Finally, as an exploratory study, the current work focused on profiling and identifying differentially expressed miRNAs, without performing functional analyses to confirm their biological roles.

## 4. Materials and Methods

The study was conducted at the University Hospital of Goethe University Frankfurt, Germany, following approval by the local Institutional Review Board (approval number 89/19). Written informed consent was obtained from all participants or, where applicable, from their legal representatives. A retrospective analysis of prospectively collected data was performed in compliance with the Declaration of Helsinki and in accordance with the STROBE reporting guidelines [54,55].

### 4.1. Study Design and Prospective Biodata Collection

For the initial screening of potential miRNAs marker candidates (Figure 1, Screening) 15 severely injured trauma patients (ISS ≥ 16) with age ≥18 years with severe isolated TBI ([TBI], AIShead ≥ 4, all other AIS ≤ 1) and 15 age- and sex-matched healthy volunteers (controls) were included. In the second phase (Figure 1, Validation), 25 severely injured trauma patients (ISS ≥ 16) with age ≥18 years, grouped into PT patients without TBI ([PT], AISHead = 0; *n* = 13) and patients with severe isolated TBI ([TBI], AISHead ≥ 4, all other AIS ≤ 1; *n* = 12) were included. Healthy volunteers ([Healthy], *n* = 12) served as the control group. Blood samples were collected from patients admitted to the emergency department of our Level 1 Trauma Centre at the University Hospital of the Goethe University Frankfurt am Main within 3 h (time of admission to the emergency room [ER]) and 48 h after trauma. Blood was collected following the standard hospital procedures in pre-chilled ethylenediaminetetraacetic acid (EDTA) tubes. The samples were centrifuged at 3000× *g* for 15 min at 4 °C and the supernatant (plasma) was stored at −80 °C until analysis. IL-6 (normal < 7.0 pg/mL) and IL-10 (normal < 7.0 pg/mL) concentrations were measured by IL-6/IL-10 ELISA (Eli-pair, Sino Biological, Eschborn, Germany) according to the manufacturer’s instructions. The concentrations of the clinically used biomarkers were retrieved from the routine blood count data in the patient records (department data, further information in Appendix A). Patient clinical data were obtained from the German TraumaRegistry DGU^®^ (Deutsche Gesellschaft für Unfallchirurgie, https://www.traumaregister-dgu.de accessed on 7 May 2026).

### 4.2. Identification of Potential miRNA Marker Candidates, NGS

For NGS analysis, samples were allocated into three pools per group, with each pool containing plasma from five TBI patients (mean age 52 years; 40% female) or controls (mean age 45 years; 40% female). This resulted in a total of nine pools: TBI ER, TBI 48 h, and control (*n* = 3 pools per group). For each pool, 300 µL of plasma from each individual sample was combined to generate a pooled plasma sample. From each pooled sample, 360 µL of plasma was used for direct miRNA isolation, and 720 µL was used for extracellular EV isolation followed by EV-miRNA isolation.

For EV isolation, plasma was cleared via a 30 min centrifugation at 16,000 *g* and 4 °C. EVs were isolated via size exclusion chromatography (SEC) (EX03-50, Cell guidance system, Cambridge, UK) according to the manufacturer’s protocol. In brief, 100 µL of plasma were first clarified by centrifugation (16,000 *g*, 4 °C, 1 h) and then loaded onto a PBS-washed column. EVs were eluted with 180 µL of 0.2 µm filtered PBS. EV isolates were characterized via particle size analysis (NTA, Nanosight NS500, Malvern Panalytical, Kassel, Germany) and marker expression, as described previously [36,52] (Supplementary Figure S1).

miRNAs were isolated from plasma or EVs by means of miRNeasy Serum/Plasma Advanced Kit (Qiagen Inc., Hilden, Germany) according to the manufacturer’s protocol. Isolated plasma- and EV-derived miRNAs were transported on dry ice to the GenXpro GmbH (Frankfurt, Germany) for NGS. Sequencing and bioinformatic analysis were performed as described in detail elsewhere [34,35]. Candidate miRNAs were selected based on the expression differences observed in the screening analysis and on evidence from the literature linked to neuroinflammation and traumatic (brain) injury.

### 4.3. Enrichment Analysis

MicroRNA network construction, Reactome pathway analysis and Gene Ontology biological process (GO: BP) enrichment analysis were performed using miRNet (https://www.mirnet.ca, accessed on 30 February 2026). Analyses were conducted separately for the sets of up- and downregulated miRNAs.

### 4.4. Marker Validation: RT-ddPCR miRNA Expression Analysis

To validate the differential expression of selected miRNAs, their expression levels were first compared between TBI patients and controls using reverse transcription droplet digital PCR (RT-ddPCR). miRNAs showing significant differential expression were subsequently validated in the TBI and PT cohorts.

miRNAs were isolated from individual plasma samples (100 µL) or from EVs (180 µL) derived from individual plasma samples, as described previously. cDNA synthesis was performed using 6.5 µL of RNA with the miRCURY LNA RT Kit (Qiagen, Hilden, Germany), according to the manufacturer’s instructions. To monitor reverse transcription efficiency, 0.5 µL of the spike-in control cel-miR-39-3p was added to each reaction. For ddPCR analysis, cDNA samples were diluted 1:500 for plasma-derived samples or 1:50 for EV-derived samples in RNase-free water.

Each 20 µL ddPCR reaction contained 9 µL of diluted cDNA, 10 µL of 2× QX200 ddPCR EvaGreen Supermix (Bio-Rad Laboratories, Hercules, CA, USA), and 1 µL of the corresponding miRCURY LNA miRNA PCR Assay (Qiagen). Droplets were generated by adding 70 µL of EvaGreen droplet generation oil and processed using the QX200 Droplet Generator (Bio-Rad Laboratories).

PCR amplification was carried out in a PTC Tempo Deep Well Thermal Cycler (Bio-Rad Laboratories) under the following conditions: 95 °C for 5 min; 40 cycles of 95 °C for 30 s (ramp rate 2 °C/s) and 51 °C for 1 min (ramp rate 2 °C/s); followed by 5 min at 4 °C and 5 min at 90 °C. Droplets were read using the QX200 Droplet Reader, and data were analyzed with QuantaSoft Analysis Pro software (version 2.1, Bio-Rad Laboratories). Results are reported as copies/µL normalized to the spike-in control cel-miR-39.

### 4.5. Statistical Analysis

Continuous variables with a normal distribution are presented as mean ± standard deviation (SD), whereas non-normally distributed continuous variables and categorical data are reported as median with interquartile range (IQR). Group comparisons for categorical variables were performed using Fisher’s exact test, while continuous variables were analyzed using the Mann–Whitney U test for two-group comparisons and the Kruskal–Wallis test for three-group comparisons. Post hoc pairwise comparisons were adjusted using the Bonferroni correction. To account for potential confounding due to baseline differences between study groups, multivariable analyses were additionally performed using multiple linear regression. For each miRNA, expression levels were modeled as the dependent variable, with group (TBI, PT), age, ISS, and ASA classification included as independent variables. Regression coefficients (β), standard errors, 95% confidence intervals, and *p*-values were reported for each covariate. Correlations between miRNA expression levels and injury-related parameters were assessed using Spearman’s rank correlation coefficient. A two-sided *p*-value < 0.05 was considered statistically significant. Given the exploratory nature of the study and the number of miRNAs analyzed, no additional correction for multiple testing across all miRNAs was applied; therefore, findings should be interpreted as hypothesis-generating. All analyses were performed using SPSS version 29 (IBM Corp., Chicago, IL, USA), and graphs were generated using GraphPad Prism 10 (version 10.4.2.(633), GraphPad Software, San Diego, CA, USA).

## 5. Conclusions

This study shows that circulating miRNA changes after injury are mainly driven by overall trauma severity rather than brain injury specifically. Most plasma miRNAs reflected the general injury burden and were not independent of clinical factors such as ISS. In contrast, EV-associated miRNAs showed more varied patterns, with miR-1469 emerging as a candidate independently associated with TBI group status, suggesting that EV miR-1469 may represent a potential TBI-related signal requiring further validation.

## Figures and Tables

**Table 1:** Functional enrichment of differentially expressed miRNAs: Reactome pathways identified via miRNet.

Time Point and Regulation		Enriched Pathways (Adj *p* Value < 0.05, Top 10)
PLASMA		
ER	Up	Cellular responses to stress; pre-NOTCH transcription and translation; cellular senescence; signaling by PDGF; oxidative stress-induced senescence; intrinsic pathway for apoptosis; signaling by NGF
	Down	Gene expression; cellular responses to stress; cellular senescence; oxidative stress-induced senescence; oncogene-induced senescence; signaling by TGF-beta receptor complex; signaling by SCF-KIT; pre-NOTCH transcription and translation
48 h	Up	ns
	Down	Gene expression; cellular responses to stress; cellular senescence; pre-NOTCH transcription and translation; pre-NOTCH expression and processing; oxidative stress-induced senescence; signaling by NOTCH; oncogene-induced senescence; generic transcription pathway; signaling by SCF-KIT
EVs		
ER	Up	Translocation of GLUT4 to the plasma membrane; VEGFR2-mediated cell proliferation; downstream signal transduction; downstream signaling of activated FGFR1, FGFR2, FGFR3, FGFR4; VEGFA-VEGFR2 pathway; intrinsic pathway for apoptosis; activation of BAD and translocation to mitochondria
	Down	Pre-NOTCH transcription and translation; oncogene-induced senescence; Caspase activation via extrinsic apoptotic signaling pathway; pre-NOTCH expression and processing; activated TLR4 signaling; signaling by NOTCH; toll-like receptor 4 (TLR4) Cascade; apoptosis; ligand-dependent caspase activation; signaling by TGF-beta receptor complex
48 h	Up	ns
	Down	Cellular responses to stress; cellular senescence; pre-NOTCH transcription and translation; gene expression; oncogene-induced senescence; pre-NOTCH expression and processing; signaling by NOTCH; oxidative stress-induced senescence; signaling by SCF-KIT; signaling by the B cell receptor (BCR)

ns—no significantly enriched pathways were found.

**Table 2:** Correlation analysis of miRNA expression levels and clinical data.

miRNA/Time Point		Parameter (Time Point)	Spearman r	*p*-Value
Plasma	miR-142-3p 48 h	RISC2	−0.52	0.02
		Base excess (48 h)	−0.45	0.04
		Thrombocytes (ICU)	0.49	0.02
	miR-4433b-5p 48 h	Thrombocytes (ICU)	0.45	0.03
		AST (24 h)	0.43	0.04
		GLDH (24 h)	0.50	0.01
		Creatine kinase (CK, 24 h)	0.41	0.05
		Myoglobin (24 h)	0.47	0.02
		Shock index	0.43	0.05
		Shock class	0.45	0.03
		Vasopressor (ER)	0.55	0.01
	miR-22-5p 48 h	RISC2	−0.54	0.01
		Creatine Kinase (48 h)	0.48	0.04
		Vasopressor (ER)	0.48	0.03
EVs	miR-1469 ER	Glasgow Coma Scale	0.43	0.05
		Catecholamine (24 h)	0.50	0.01
		Prothrombin time (ICU)	−0.45	0.03
		CK-MB (24 h)	0.47	0.02
		C-reactive protein (48 h)	0.76	0.04
		RBC Concentrates (ER)	0.50	0.01
	miR-3182 48 h	RISC2	−0.56	0.01
		GLDH (24 h)	0.51	0.01
		Myoglobin (24 h)	0.55	0.01
		Creatin Kinase (48 h)	0.61	0.01
	miR-1237-5p 48 h	Interleukin-6 (ER)	0.55	0.01
		GLDH (48 h)	0.66	0.02
		Vasopressor (ER)	0.65	0.002
		RBC Concentrates (ER)	0.47	0.03
	miR-6508-3p 48 h	Injury severity score	0.44	0.04
		Hemoglobin (24 h)	−0.48	0.03
		Hemoglobin (ICU)	−0.56	0.01
		Ventilation (ICU)	−0.49	0.05
		ALT (24 h)	0.45	0.04
		GLDH (24 h)	0.45	0.03
		GLDH (48 h)	0.60	0.03
		Creatine Kinase (48 h)	0.49	0.03
		RBC Concentrates (ER)	0.67	0.001

RISC 2 = Revised Injury Severity Classification II, RBC = red blood cell, ALT = Alanine-Aminotransferase, AST = Aspartate-Aminotransferase, MB = muscle–brain type, GLDH = Glutamate Dehydrogenase, ICU = Intensive Care Init.

## Data Availability

The data discussed in this publication have been deposited in NCBI’s Gene Expression Omnibus [56] and are accessible through GEO Series accession number GSE325396 (https://www.ncbi.nlm.nih.gov/geo/query/acc.cgi?acc=GSE325396, submitted on 19 March 2026).

## Supplementary Materials

The following supporting information can be downloaded at: https://www.mdpi.com/article/10.3390/ijms27104248/s1.

## Abbreviations

The following abbreviations are used in this manuscript:

AIS	Abbreviated Injury Score
ALT	Alanine-Aminotransferase
ASA	American Society of Anesthesiologists Physical Status Classification System
AST	Aspartate-Aminotransferase
BBB	Blood–brain barrier
CK	Creatine Kinase
CRP	C-reactive protein
ER	Emergency room
EV	Extracellular vesicle
GCS	Glasgow Coma Scale
GLDH	Glutamate-Dehydrogenase
ICU	Intensive care unit
IL	Interleukin
INT	Endotracheal Intubation/Ventilation
IQR	Interquartile range
ISS	Injury severity score
miRNA	microRNA
NGS	Next-generation sequencing
PT	Polytrauma
RBC	Red blood cell
RISC2	Revised Injury Severity Classification
RT-ddPCR	RT- droplet digital PCR
SI	Shock index
TBI	Traumatic brain injury

## Appendix A

Patients with known pre-existing immunological disorders, immunosuppressive medication, burns, concomitant acute myocardial infarction, thromboembolic events and patients who died within 5 days of hospital admission (incomplete serial blood samples) were excluded.

Time points: Available data from relevant preliminary studies have shown that neuronal biomarkers as well as inflammatory markers are expressed in a time-dependent manner after trauma [24,36]. In the case of primary brain injury, the first few hours play a particularly important role. In addition, the severity of injury after TBI is characterized by the development of secondary brain damage due to neuroinflammatory processes after trauma. For these reasons, in the project we included patient samples taken at early (admission to ER) and later (48 h) time points to obtain a broad list of possible miRNA biomarkers.

## References

[1] Chang H.Y.M., Flahive J., Bose A., Goostrey K., Osgood M., Carandang R., Hall W., Muehlschlegel S. (2022). Predicting mortality in moderate-severe TBI patients without early withdrawal of life-sustaining treatments including ICU complications: The MYSTIC-score. J. Crit. Care.

[2] Craig Williamson V.R. Traumatic Brain Injury: Epidemiology, Pathophysiology, and Classification. https://www.uptodate.com.

[3] Younsi A., Unterberg A., Marzi I., Steudel W.-I., Uhl E., Lemcke J., Berg F., Woschek M., Friedrich M., Clusmann H. (2023). Development and first results of a national databank on care and treatment outcome after traumatic brain injury. Eur. J. Trauma Emerg. Surg. Off. Publ. Eur. Trauma Soc..

[4] van Wessem K.J.P., Benders K.E.M., Leenen L.P.H., Hietbrink F. (2024). TBI related death has become the new epidemic in polytrauma: A 10-year prospective cohort analysis in severely injured patients. Eur. J. Trauma Emerg. Surg..

[5] Thapa K., Khan H., Singh T.G., Kaur A. (2021). Traumatic Brain Injury: Mechanistic Insight on Pathophysiology and Potential Therapeutic Targets. J. Mol. Neurosci..

[6] Price L., Wilson C., Grant G. (2016). Chapter 4 Blood–Brain Barrier Pathophysiology following Traumatic Brain Injury. Translational Research in Traumatic Brain Injury.

[7] Maas A.I.R., Menon D.K., Manley G.T., Abrams M., Åkerlund C., Andelic N., Aries M., Bashford T., Bell M.J., Bodien Y.G. (2022). Traumatic brain injury: Progress and challenges in prevention, clinical care, and research. Lancet Neurol..

[8] Slot R.E.R., Helbok R., van der Jagt M. (2025). Update on traumatic brain injury in the ICU. Curr. Opin. Anaesthesiol..

[9] Kennedy L., Nuno M., Gurkoff G.G., Nosova K., Zwienenberg M. (2022). Moderate and severe TBI in children and adolescents: The effects of age, sex, and injury severity on patient outcome 6 months after injury. Front. Neurol..

[10] Nasrallah F., Bellapart J., Walsham J., Jacobson E., To X.V., Manzanero S., Brown N., Meyer J., Stuart J., Evans T. (2023). PREdiction and Diagnosis using Imaging and Clinical biomarkers Trial in Traumatic Brain Injury (PREDICT-TBI) study protocol: An observational, prospective, multicentre cohort study for the prediction of outcome in moderate-to-severe TBI. BMJ Open.

[11] Corbella D., Zangari R., Biroli F., Magnone S., Cavalleri G., Passoni M., Martchenko S., Marchesi S., Zacchetti L., Ferri F. (2025). Comparing survival and outcomes in isolated versus polytrauma-associated TBI: A retrospective cohort study. J. Neurosurg. Sci..

[12] Schindler C., Lustenberger T. (2024). Focus on challenges and advances in the treatment of traumatic brain injury. Eur. J. Trauma Emerg. Surg. Off. Publ. Eur. Trauma Soc..

[13] Valcarcel C.R., Bieler D., Bass G.A., Gaarder C., Hildebrand F. (2025). ESTES recommendations for the treatment of polytrauma-a European consensus based on the German S3 guidelines for the treatment of patients with severe/multiple injuries. Eur. J. Trauma Emerg. Surg..

[14] Niessen P.D.M., Krijnen P., Leijdesdorff H.A., Peul W.C., Schipper I.B. (2025). Interpreting traumatic brain injury severity: Analysis of the correlation between Glasgow coma scale and abbreviated injury scale. Eur. J. Trauma Emerg. Surg..

[15] Korley F.K., Jain S., Sun X., Puccio A.M., Yue J.K., Gardner R.C., Wang K.K.W., Okonkwo D.O., Yuh E.L., Mukherjee P. (2022). Prognostic value of day-of-injury plasma GFAP and UCH-L1 concentrations for predicting functional recovery after traumatic brain injury in patients from the US TRACK-TBI cohort: An observational cohort study. Lancet Neurol..

[16] Pelinka L.E., Kroepfl A., Leixnering M., Buchinger W., Raabe A., Redl H. (2004). GFAP versus S100B in serum after traumatic brain injury: Relationship to brain damage and outcome. J. Neurotrauma.

[17] Mendes Arent A., de Souza L.F., Walz R., Dafre A.L. (2014). Perspectives on molecular biomarkers of oxidative stress and antioxidant strategies in traumatic brain injury. BioMed Res. Int..

[18] Schindler C.R., Lustenberger T., Woschek M., Störmann P., Henrich D., Radermacher P., Marzi I. (2020). Severe Traumatic Brain Injury (TBI) Modulates the Kinetic Profile of the Inflammatory Response of Markers for Neuronal Damage. J. Clin. Med..

[19] Ghosh S., Kumar V., Mukherjee H., Lahiri D., Roy P. (2021). Nutraceutical regulation of miRNAs involved in neurodegenerative diseases and brain cancers. Heliyon.

[20] Mahmoud M.M., Sanad E.F., Hamdy N.M. (2021). MicroRNAs’ role in the environment-related non-communicable diseases and link to multidrug resistance, regulation, or alteration. Environ. Sci. Pollut. Res. Int..

[21] Dykxhoorn D.M., Novina C.D., Sharp P.A. (2003). Killing the messenger: Short RNAs that silence gene expression. Nat. Rev. Mol. Cell Biol..

[22] Nguyen T.P.N., Kumar M., Fedele E., Bonanno G., Bonifacino T. (2022). MicroRNA Alteration, Application as Biomarkers, and Therapeutic Approaches in Neurodegenerative Diseases. Int. J. Mol. Sci..

[23] Pan Y.-B., Sun Z.-L., Feng D.-F. (2017). The Role of MicroRNA in Traumatic Brain Injury. Neuroscience.

[24] Liu X., Zhang L., Cao Y., Jia H., Li X., Li F., Zhang S., Zhang J. (2022). Neuroinflammation of traumatic brain injury: Roles of extracellular vesicles. Front. Immunol..

[25] Pedder J.H., Sonabend A.M., Cearns M.D., Michael B.D., Zakaria R., Heimberger A.B., Jenkinson M.D., Dickens D. (2025). Crossing the blood-brain barrier: Emerging therapeutic strategies for neurological disease. Lancet Neurol..

[26] Tomatis F., Rosa S., Simões S., Barão M., Jesus C., Novo J., Barth E., Marz M., Ferreira L. (2024). Engineering extracellular vesicles to transiently permeabilize the blood-brain barrier. J. Nanobiotechnol..

[27] Ghaith H.S., Nawar A.A., Gabra M.D., Abdelrahman M.E., Nafady M.H., Bahbah E.I., Ebada M.A., Ashraf G.M., Negida A., Barreto G.E. (2022). A Literature Review of Traumatic Brain Injury Biomarkers. Mol. Neurobiol..

[28] Tkach M., Théry C. (2016). Communication by Extracellular Vesicles: Where We Are and Where We Need to Go. Cell.

[29] Wessler S., Meisner-Kober N. (2025). On the road: Extracellular vesicles in intercellular communication. Cell Commun. Signal..

[30] Sproviero D., Gagliardi S., Zucca S., Arigoni M., Giannini M., Garofalo M., Olivero M., Dell’Orco M., Pansarasa O., Bernuzzi S. (2021). Different miRNA Profiles in Plasma Derived Small and Large Extracellular Vesicles from Patients with Neurodegenerative Diseases. Int. J. Mol. Sci..

[31] Nobrega M., Reis M.B.D., de Souza M.F., Furini H.H., Costa Brandão Berti F., Souza I.L.M., Mingorance Carvalho T., Zanata S.M., Fuganti P.E., Malheiros D. (2025). Comparative analysis of extracellular vesicles miRNAs (EV-miRNAs) and cell-free microRNAs (cf-miRNAs) reveals that EV-miRNAs are more promising as diagnostic and prognostic biomarkers for prostate cancer. Gene.

[32] Groven R.V.M., Greven J., Mert Ü., Horst K., Zhao Q., Blokhuis T.J., Huber-Lang M., Hildebrand F., van Griensven M. (2023). Circulating miRNA expression in extracellular vesicles is associated with specific injuries after multiple trauma and surgical invasiveness. Front. Immunol..

[33] Zilliox M.J., Foecking E.M., Kuffel G.R., Conneely M., Saban K.L., Herrold A.A., Kletzel S.L., Radke J.R., Walsh E., Guernon A. (2023). An Initial miRNA Profile of Persons With Persisting Neurobehavioral Impairments and States of Disordered Consciousness After Severe Traumatic Brain Injury. J. Head Trauma Rehabil..

[34] Hörauf J.-A., Saenger M., Störmann P., El Saman A., Marzi I., Henrich D., Leppik L., Schindler C.R. (2025). Circulating microRNA Profiles in Acute Spinal Cord Injury: Evidence for Distinct Plasma Signatures Compared with Polytrauma Patients. Int. J. Mol. Sci..

[35] Han J., Leppik L., Sztulman L., de Rosa R., Pfeiffer V., Busse L.-C., Kontaxi E., Adam E., Henrich D., Marzi I. (2025). Dual Roles of Plasma miRNAs in Myocardial Injuries After Polytrauma: miR-122-5p and miR-885-5p Reflect Inflammatory Response, While miR-499a-5p and miR-194-5p Contribute to Cardiomyocyte Damage. Cells.

[36] Schindler C.R., Hörauf J.A., Weber B., Schaible I., Marzi I., Henrich D., Leppik L. (2024). Identification of novel blood-based extracellular vesicles biomarker candidates with potential specificity for traumatic brain injury in polytrauma patients. Front. Immunol..

[37] Keller A., Gröger L., Tschernig T., Solomon J., Laham O., Schaum N., Wagner V., Kern F., Schmartz G.P., Li Y. (2022). miRNATissueAtlas2: An update to the human miRNA tissue atlas. Nucleic Acids Res..

[38] Cui S., Chen Y., Guo Y., Wang X., Chen D. (2023). Hsa-miR-22-3p inhibits liver cancer cell EMT and cell migration/ invasion by indirectly regulating SPRY2. PLoS ONE.

[39] Sharma S. (2017). Immunomodulation: A definitive role of microRNA-142. Dev. Comp. Immunol..

[40] Sun T.-Y., Chen X.-R., Liu Z.-L., Zhao L.-L., Jiang Y.-X., Qu G.-Q., Wang R.-S., Huang S.-Z., Liu L. (2014). Expression profiling of microRNAs in hippocampus of rats following traumatic brain injury. J. Huazhong Univ. Sci. Technol. Med. Sci..

[41] Zheng P., Ren D., Yu C., Zhang X., Zhang Y. (2022). DNA Methylation-Related circRNA_0116449 Is Involved in Lipid Peroxidation in Traumatic Brain Injury. Front. Mol. Neurosci..

[42] Xie Z., Liu L., Guo Y., Jiang H., Li L., Qiao Z., Wang J. (2025). Exosomal miR-432-5p, miR-4433b-5p, and miR-599: Biomarkers for Monitoring the Severity of Anti-N-methyl-D-aspartate Receptor Encephalitis. J. Integr. Neurosci..

[43] Nicoletti A.d.S., Visacri M.B., Da Ronda C.R.S.C., Vasconcelos P.E.d.N.S., Quintanilha J.C.F., de Souza R.N., Ventura D.d.S., Eguti A., Silva L.F.d.S., Perroud Junior M.W. (2022). Differentially expressed plasmatic microRNAs in Brazilian patients with Coronavirus disease 2019 (COVID-19): Preliminary results. Mol. Biol. Rep..

[44] Carvalho T.M., Brasil G.O., Jucoski T.S., Adamoski D., de Lima R.S., Spautz C.C., Anselmi K.F., Ozawa P.M.M., Cavalli I.J., Carvalho de Oliveira J. (2022). MicroRNAs miR-142-5p, miR-150-5p, miR-320a-3p, and miR-4433b-5p in Serum and Tissue: Potential Biomarkers in Sporadic Breast Cancer. Front. Genet..

[45] Tan M.S., Cheah P.-L., Chin A.-V., Looi L.-M., Chang S.-W. (2023). Differential Expression Analysis of Blood MicroRNA in Identifying Potential Genes Relevant to Alzheimer’s Disease Pathogenesis, Using an Integrated Bioinformatics and Machine Learning Approach. Appl. Sci..

[46] Francisco L.F.V., Birolli W.G., Hirai W., Nunes C.R., Gonçalves I.Z., Vazquez F.d.L., Dos Santos Neto Á.J., Barbosa Junior F., Marques M.M.C., Silveira H.C.S. (2025). Analysis of the expression profile and biological function of plasma miRNAs in chronic lymphocytic leukemia and multiple myeloma patients occupationally exposed to pesticides. Ecotoxicol. Environ. Saf..

[47] DiVincenzo M.J., Barricklow Z., Schwarz E., Moufawad M., Howard J.H., Yu L., Chung C., Gru A.A., Carson W.E. (2021). Loss of miR-1469 expression mediates melanoma cell migration and invasion. PLoS ONE.

[48] Wei Y., Zhou T., Pan R., Nie X., Liu Z., Shi Z., Zeng Y., Zhang R., Deng Y., Li D. (2024). Exosomes containing miR-1469 regulate natural killer cells by targeting CD122 in non-segmental vitiligo. J. Dermatol. Sci..

[49] Funatsuki T., Ogata H., Tahara H., Shimamoto A., Takekita Y., Koshikawa Y., Nonen S., Higasa K., Kinoshita T., Kato M. (2023). Changes in Multiple microRNA Levels with Antidepressant Treatment Are Associated with Remission and Interact with Key Pathways: A Comprehensive microRNA Analysis. Int. J. Mol. Sci..

[50] Kumar S., Orlov E., Gowda P., Bose C., Swerdlow R.H., Lahiri D.K., Reddy P.H. (2022). Synaptosome microRNAs regulate synapse functions in Alzheimer’s disease. npj Genom. Med..

[51] Schindler C.R., Woschek M., Vollrath J.T., Kontradowitz K., Lustenberger T., Störmann P., Marzi I., Henrich D. (2020). miR-142-3p Expression Is Predictive for Severe Traumatic Brain Injury (TBI) in Trauma Patients. Int. J. Mol. Sci..

[52] Weber B., Henrich D., Schindler C.R., Marzi I., Leppik L. (2023). Release of exosomes in polytraumatized patients: The injury pattern is reflected by the surface epitopes. Front. Immunol..

[53] Hörauf J.-A., Leppik L., Weber B., Hildebrand F., Störmann P., Henrich D., Marzi I., Schindler C.R. (2025). MiR338-3p expression in extracellular vesicles after severe trauma with or without traumatic brain injury. Brain Commun..

[54] von Elm E., Altman D.G., Egger M., Pocock S.J., Gøtzsche P.C., Vandenbroucke J.P. (2008). Das Strengthening the Reporting of Observational Studies in Epidemiology (STROBE-) Statement. Internist.

[55] Benchimol E.I., Smeeth L., Guttmann A., Harron K., Moher D., Petersen I., Sørensen H.T., von Elm E., Langan S.M. (2015). The REporting of studies Conducted using Observational Routinely-collected health Data (RECORD) statement. PLoS Med..

[56] Edgar R., Domrachev M., Lash A.E. (2002). Gene Expression Omnibus: NCBI gene expression and hybridization array data repository. Nucleic Acids Res..

