# Supplementary material for: Extracellular Vesicle and Plasma miRNAs as Candidate Biomarkers of Traumatic Brain Injury in the Context of Polytrauma
Source: Int J Mol Sci. 2026 May 10;27(10):4248. doi: 10.3390/ijms27104248 (PMC13206937; doi:10.3390/ijms27104248)
Supplement: Supplementary file 1 [file ijms-27-04248-s001.zip › ijms-4240502-supplementary.pdf]

Supplementary table S1. Demographic and clinical data of patients' cohort

Demographic and clinical data	**TBI** (n= 12)	**PT** (n= 13)
Sex (male:female)	2:10	4:9
Age (years)	66 (59 – 76)	40 (32 – 64)
ASA	2 (2 – 2)	1 (1 – 2)
Injury Severity Score (ISS)	19 (16 – 25)	25 (21 – 29)
AIS_{Head}	4 (4 – 5)	0
AIS_{Thorax}	0	3 (2 – 4)
$AIS_{Abdomen}$	0	2 (1 – 3)
$AIS_{Extremities}$	0	3 (2 – 4)
Glasgow Coma Scale (GCS)	9 (5 – 13)	15 (14 – 15)

American Society of Anesthesiologists (ASA) Classification. Abbreviated Injury Scale (AIS).

Supplementary table S2. Biological process enrichment analysis using miRNet

(Top 10, adj p<0.05)

Time Point	Regulation	GO: Biological Processes
		PLASMA
ER	Up	Regulation of Cellular Protein Metabolic Process; Regulation of Protein Metabolic Process; Protein Phosphorylation; Negative Regulation of Programmed Cell Death; Negative Regulation of Apoptotic Process; Cell Proliferation
	Down	Negative Regulation of Transcription from RNA Polymerase II Promoter; Regulation of Translation; Negative Regulation of Cellular Metabolic Process; Regulation of Cell Cycle; Negative Regulation of Metabolic Process; Negative Regulation of Biosynthetic Process; Regulation of Protein Kinase Activity; Positive Regulation of Cellular Component Organization
48h	Up	Negative Regulation of Transcription from RNA Polymerase II Promoter; Negative Regulation of Cellular Metabolic Process; Negative Regulation of Nucleobase-Containing Compound Metabolic Process; Negative Regulation of Transcription DNA-Dependent;<br>Negative Regulation of RNA Metabolic Process; DNA Damage Response, Signal Transduction by P53 Class Mediator; Negative Regulation of Cellular Biosynthetic Process; Signal Transduction in Response To DNA Damage
	Down	Negative Regulation of Cellular Metabolic Process; Negative Regulation of Transcription from RNA Polymerase II Promoter; Negative Regulation of Cellular Biosynthetic Process; Negative Regulation of Metabolic Process; Negative Regulation of Transcription, DNA-Dependent; Negative Regulation of RNA Metabolic Process; Negative Regulation of Nucleobase-Containing Compound Metabolic Process; Regulation of Translation; Regulation of Transcription from RNA Polymerase II Promoter
		EVs
ER	Up	Negative Regulation of Apoptotic Process; Negative Regulation of Programmed Cell Death; Negative Regulation of Cellular Metabolic Process; Negative Regulation of RNA Metabolic Process; Regulation of Cell Differentiation; Positive Regulation of Cell Differentiation; Negative Regulation of Metabolic Process; Negative Regulation of Nucleobase-Containing Compound Metabolic Process; Positive Regulation of Cell Migration
	Down	Chromatin Modification; Positive Regulation of Cellular Component Organization; Positive Regulation of Transcription from RNA Polymerase II Promoter; Negative Regulation of Cellular Metabolic Process; Covalent Chromatin Modification;

		Histone Modification; Negative Regulation of Metabolic Process; Myeloid Cell Differentiation; Negative Regulation of Apoptotic Process; Tube Development
48h	Up	Organic Acid Transport; Carboxylic Acid Transport; Anion Transport; Activation of Jun Kinase Activity; Regulated Secretory Pathway; Superoxide Metabolic Process; Bone Remodeling; Positive Regulation of Cytokine Secretion; Positive Regulation of Jun Kinase Activity; Positive Regulation of Cytokine Biosynthetic Process
	Down	Negative Regulation of Cellular Biosynthetic Process; Negative Regulation of Biosynthetic Process;<br>Negative Regulation of Metabolic Process; Interphase of Mitotic Cell Cycle; Negative Regulation of Cellular Metabolic Process; Interphase;<br>Negative Regulation of Transcription from RNA Polymerase II Promoter; Negative Regulation of Transcription, DNA-Dependent; Regulation of Translation; Negative Regulation of RNA Metabolic Process

Supplementary Table S3. Multiple linear regression of selected miRNA expression levels adjusted for study group, age, ISS, and ASA classification.

miRNA	Parameter estimates	Variable	Estimate	Standard error	95% CI (asymptotic)	|t|	P value	P value summary
miR-22-5p	β0	Intercept	-0,07984	0,04998	-0,1849 to 0,02516	1,597	0,1276	ns
	β1	X1 (Group)	-0,02721	0,04113	-0,1136 to 0,05921	0,6615	0,5167	ns
	β2	X2 (Age)	0,0001718	0,001074	-0,002084 to 0,002427	0,16	0,8746	ns
	β3	X3 (ISS)	0,006916	0,001781	0,003173 to 0,01066	3,882	0,0011	**
	β4	X4 (ASA)	-0,01918	0,02622	-0,07426 to 0,03590	0,7316	0,4739	ns
miR-142-3p	β0	Intercept	0,1568	3,21	-6,587 to 6,900	0,04884	0,9616	ns
	β1	X1 (Group)	-2,929	2,642	-8,478 to 2,621	1,109	0,2822	ns
	β2	X2 (Age)	-0,02063	0,06895	-0,1655 to 0,1242	0,2992	0,7682	ns
	β3	X3 (ISS)	0,2371	0,1144	-0,003252 to 0,4775	2,072	0,0529	ns
	β4	X4 (ASA)	-0,133	1,684	-3,670 to 3,404	0,079	0,9379	ns
miR-4433b-5	β0	Intercept	0,053	0,03713	-0,02500 to 0,1310	1,428	0,1706	ns
	β1	X1 (Group)	-0,01945	0,03056	-0,08365 to 0,04475	0,6365	0,5325	ns
	β2	X2 (Age)	-0,001803	0,0007976	-0,003479 to -0,0001272	2,26	0,0364	*
	β3	X3 (ISS)	0,004356	0,001323	0,001576 to 0,007137	3,292	0,0041	**
	β4	X4 (ASA)	0,00337	0,01948	-0,03755 to 0,04429	0,173	0,8646	ns
	β0	Intercept	0,0429	0,0315	-0,02281 to 0,1086	1,362	0,1884	ns
EV miR-1469	β1	X1 (Group)	-0,07163	0,02559	-0,1250 to -0,01825	2,799	0,0111	*
	β2	X2 (Age)	0,0008895	0,0006397	-0,0004448 to 0,002224	1,391	0,1796	ns
	β3	X3 (ISS)	-0,001449	0,00112	-0,003786 to 0,0008881	1,293	0,2107	ns
	β4	X4 (ASA)	0,009327	0,01601	-0,02407 to 0,04272	0,5826	0,5667	ns
EV miR-3182	β0	Intercept	-0,0004301	0,005675	-0,01235 to 0,01149	0,07579	0,9404	ns
	β1	X1 (Group)	-0,003752	0,004856	-0,01395 to 0,006449	0,7728	0,4497	ns
	β2	X2 (Age)	-0,00008017	0,0001183	-0,0003287 to 0,0001684	0,6777	0,5066	ns
	β3	X3 (ISS)	0,0001085	0,0001936	-0,0002983 to 0,0005153	0,5604	0,5821	ns
	β4	X4 (ASA)	0,003646	0,002999	-0,002654 to 0,009947	1,216	0,2397	ns
EV miR-1237	β0	Intercept	-0,006376	0,005298	-0,01751 to 0,004753	1,204	0,2443	ns
	β1	X1 (Group)	-0,003032	0,004533	-0,01256 to 0,006491	0,669	0,512	ns
	β2	X2 (Age)	-0,00008869	0,0001104	-0,0003207 to 0,0001433	0,8031	0,4324	ns
	β3	X3 (ISS)	0,0006106	0,0001808	0,0002308 to 0,0009903	3,378	0,0034	**
	β4	X4 (ASA)	0,002768	0,0028	-0,003114 to 0,008649	0,9887	0,3359	ns
EV miR-6508	β0	Intercept	0,0003376	0,004997	-0,01016 to 0,01084	0,06756	0,9469	ns
	β1	X1 (Group)	-0,002654	0,004276	-0,01164 to 0,006329	0,6206	0,5426	ns
	β2	X2 (Age)	-0,0001004	0,0001042	-0,0003193 to 0,0001184	0,964	0,3478	ns
	β3	X3 (ISS)	0,0001102	0,0001705	-0,0002480 to 0,0004684	0,6463	0,5263	ns
	β4	X4 (ASA)	0,003322	0,002641	-0,002226 to 0,008870	1,258	0,2244	ns

A

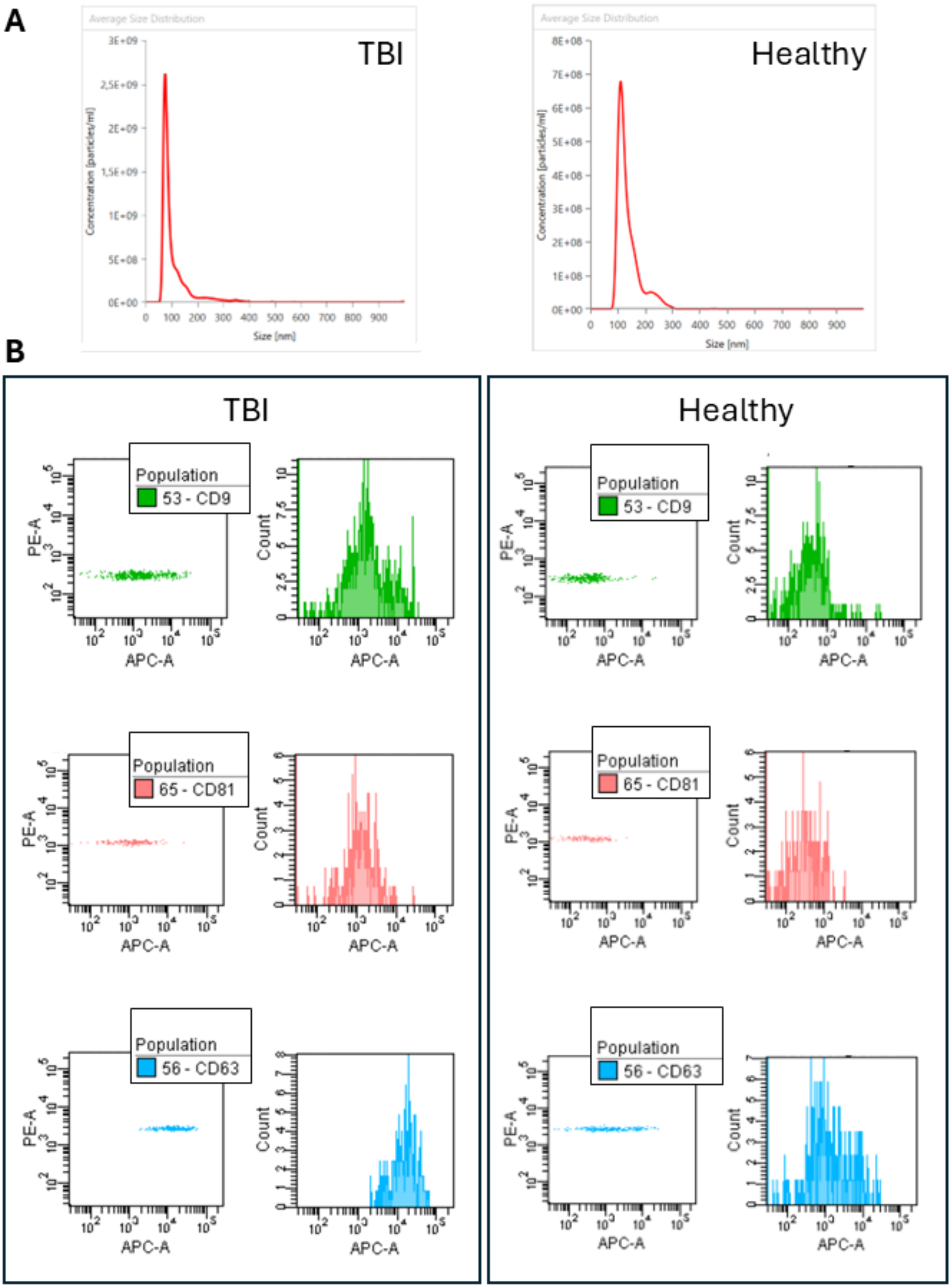

Supplementary figure S1. Representative characterization of extracellular vesicles using nanoparticle tracking analysis (NTA) and multiplex flow cytometry.

(A) Concentration and size distribution of EV particles in two representative isolates. Nanoparticle tracking analysis (NTA) was performed on 1:100 diluted samples under constant flow conditions using a Nanosight Pro device with NS XPLORER v1.1.0.6 software (Malvern Panalytical, Kassel, Germany).

(B) Detection of EV surface markers CD9, CD81, and CD63 in representative EV isolates from a TBI patient (left) and a healthy control (right). EV surface antigens were analyzed using a bead-based multiplex flow cytometry assay (MACSPlex EV Kit Neuro, Miltenyi, Bergisch Gladbach, Germany). Briefly, EV isolates (TBI and healthy control; 20 µg protein each) were incubated with fluorescently labeled beads conjugated to antibodies against specific surface epitopes and subsequently analyzed by flow cytometry (BD FACSCanto II, Becton Dickinson (BD) GmbH, Heidelberg, Germany). Data were analyzed using BD FACSDiva v6.1.3 software.
